# Pulmonary tumor thrombotic microangiopathy: Two case reports and literature review

**DOI:** 10.1097/MD.0000000000038618

**Published:** 2024-06-28

**Authors:** Hua Ma, Jian-Mei Gao, Jing Wang, Ling-Yan Huang, Xing-Cang Tian, Zhi-Gang Tian, Shao-Jin Wang, Gang Ma, Hai Tan, Shu-Xiang Zhang

**Affiliations:** a Clinical Medical College, Ningxia Medical University, Yinchuan, China; b Department of Respiratory and Critical Care Medicine, Ningxia Medical University General Hospital, Yinchuan, China; c Department of Pathology, General Hospital of Ningxia Medical University, Yinchuan, China; d Department of Radiology, General Hospital of Ningxia Medical University, Yinchuan, China.

**Keywords:** dyspnea, gastric cancer, heart failure, metastatic carcinoma, pulmonary tumor thrombotic microangiopathy, respiratory failure, tumor-related pulmonary hypertension

## Abstract

**Rationale:**

Pulmonary tumor thrombotic microangiopathy (PTTM) is a rare but serious complication in patients with malignancy; its main manifestation includes acute pulmonary hypertension with severe respiratory distress. More than 200 cases have been reported since it was first identified in 1990. PTTM accounts for approximately 0.9% to 3.3% of deaths due to malignancy, but only a minority of patients are diagnosed ante-mortem, with most patients having a definitive diagnosis after autopsy.

**Patient concerns:**

Two middle-aged women both died within a short period of time due to progressive dyspnea and severe pulmonary hypertension.

**Diagnoses:**

One patient was definitively confirmed as a gastrointestinal malignant tumor by liver puncture biopsy pathology. Ultimately, the clinical diagnosis was pulmonary tumor thrombotic microangiopathy.

**Interventions:**

The patient was treated symptomatically with oxygen, diuresis, and anticoagulation, while a liver puncture was perfected to clarify the cause.

**Outcomes:**

Two cases of middle-aged female patients with rapidly progressive pulmonary hypertension and respiratory failure resulted in death with malignant neoplasm.

**Lessons:**

PTTM has a rapid onset and a high morbidity and mortality rate. Our clinicians need to be more aware of the need for timely diagnosis through a targeted clinical approach, leading to more targeted treatment and a better prognosis.

## 1. Introduction

Pulmonary tumor thrombotic microangiopathy (PTTM) is a rare complication of malignancy, the most prominent of which is severe pulmonary hypertension, a specific microangiopathy associated with pulmonary intimal myofibroblast hyperplasia, pulmonary tumors thrombosis. It is a rare cause of rapidly progressive dyspnea in patients with confirmed or undiagnosed malignancies.^[1]^ Because PTTM is rare but rapidly progressive, ante-mortem diagnosis remains a clinical challenge.^[2–6]^ It is usually diagnosed during an autopsy.

The histological features of PTTM include fibroblast proliferation in the intima of small blood vessels and remodeling of the pulmonary vasculature, intravascular invasion by tumor cells leading to activation of the coagulation system and secondary thrombosis, and proliferation of fibroblasts and fibromuscular in the intima leading to luminal narrowing, thereby increasing pulmonary vascular resistance. Therefore, progressive exacerbation of pulmonary hypertension leads to acute or subacute pulmonary heart disease and respiratory failure.^[7]^

According to relevant literature, PTTM is associated with adenocarcinoma, most commonly gastric adenocarcinoma.^[8]^ Other cancers include lung, breast, tongue, hepatocellular, colorectal, and prostate cancers.^[9]^ A review of previous case reports showed that the most common symptoms of PTTM included cough, sputum production, shortness of breath, dyspnea, inability to lie down at night, chest tightness, chest pain, hemoptysis, fever, malaise, and wasting.^[10]^ Laboratory tests revealed the elevated levels of D-dimer (D-D), fibrin degradation products (PDF), but the platelet count was normal or reduced. There is a lack of specificity in imaging, with some cases reporting glassy interstitial lung changes on CT in patients with PTTM.^[4,11,12]^

This study reports 2 cases of PTTM with an unexplained onset of severe dyspnea and pulmonary hypertension, a short period of time to refine the clinical diagnosis upon examination, but rapidly progressive heart and respiratory failure leading to death with no chance to get treatment.

## 2. Case presentation

### 2.1. Case 1

**Figure 1.**
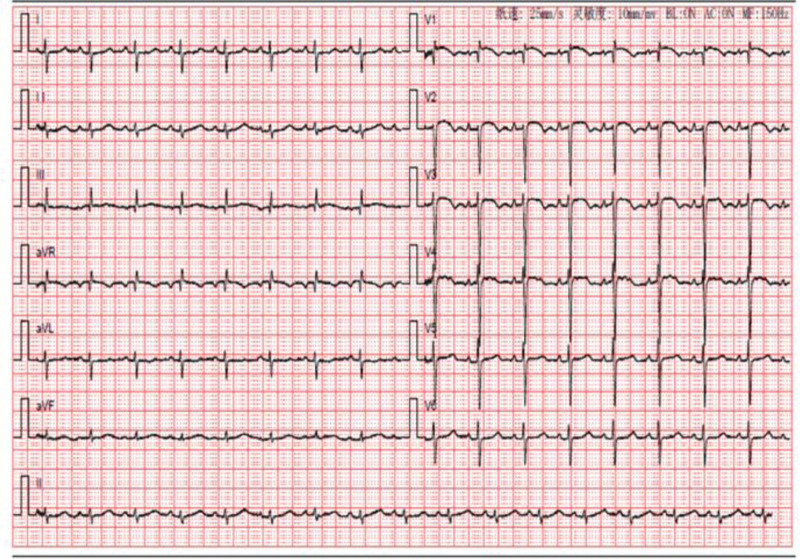
ECG on hospital admission revealed. (1) Sinus rhythm. (2) Incomplete right bundle branch block. (3) Poor R-wave increase of V1-V3 lead. (4) ST elevation of V1 lead. (5) Right axis deviation. (6) Cis-clockwise transposition. ECG = electrocardiography.

**Figure 2.**
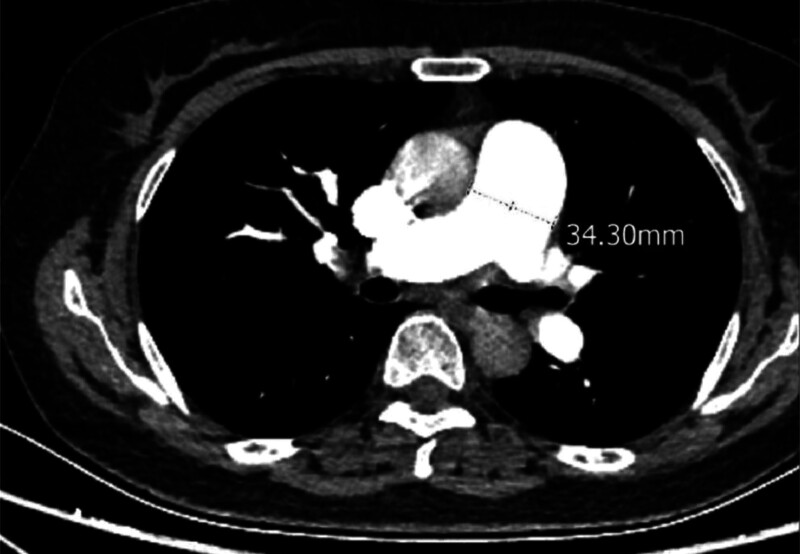
CT angiography of pulmonary arteries revealed the diameter of the pulmonary artery was 34.30 mm.

A 58-year-old woman was admitted to our hospital on April 7, 2022, with progressive worsening of chest tightness and shortness of breath for more than 1 month. Laboratory findings were as follows: WBC, 12.31 × 10^9^/L; NEUT (%), 80.8%; Hb, 132.0 g/L; platelet count, 131 × 10^9^/L; APTT, 24.7 s; D-D, 9.62 µg/mL; and N-terminal pro-brain natriuretic peptide (NT-pro BNP), 9730.60 pg/mL. Arterial blood gas analysis were as follows: PH (T), 7.417; PCO_2_ (T), 28.20 mm Hg; and PO_2_ (T), 86.20% (continuous intranasal high-flow oxygen, oxygen concentration 55%). Tumor markers were carcinoembryonic antigen, 13.40 ng/mL; glycoantigen 125, 95.50 U/mL; and glycoantigen 153, 28.20 U/mL. Electrocardiography (ECG) revealed sinus rhythm, incomplete right bundle branch block, poor R-wave increase of V1-V3 lead, ST elevation of V1 lead, right axis deviation, and cis-clockwise transposition (Fig. 1). Ultrasound cardiogram (UCG) revealed increased right atrial and right ventricular proportions, a widened main pulmonary artery, moderate mitral regurgitation, severe pulmonary hypertension (PASP, 96 mm Hg), reduced left ventricular diastolic function, and normal left ventricular systolic function (Table 1). Computed tomography pulmonary angiography (CTPA) revealed pulmonary artery widening but no signs of embolism (Fig. 2).

**Table 1:** Echocardiogram parameters.

Parameters	Result	Reference value
EF	65.61%	>50%
AAO	29	28–38 mm
AO	19	20–35 mm
LA	35	19–35 mm
RA	49*52	33–41 mm
PA	29	12–26 mm
RVD	30	7–23 mm
TV	4.9	0.3–0.7 m/s

AAO = ascending aorta, AO = aorta, EF = ejection fraction, LA = left ventricle, PA = pulmonary artery, RA = right atrium, RVD = right ventricle diameter, TV = tricuspidvalve velocity.

**Figure 3.**
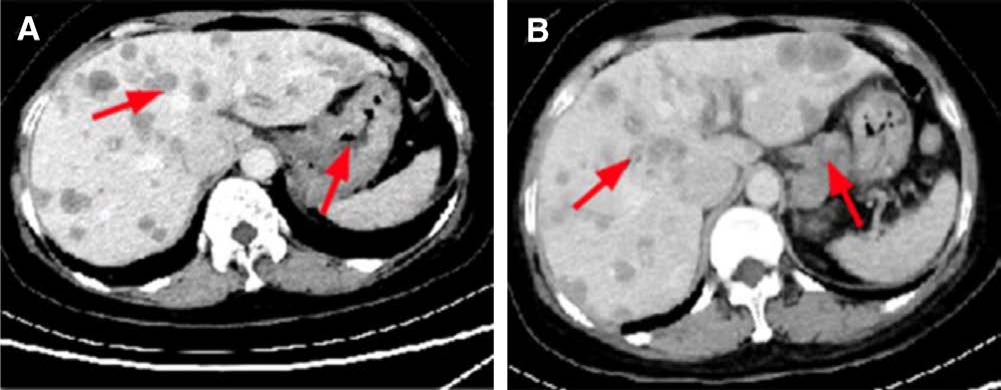
Abdominal enhanced CT. (A) Thickening of the stomach wall, a tumor with multiple metastases to the liver (arrow). (B) Multiple metastases to the liver and lymph node metastases to the side of the lesser curvature of the stomach (arrow).

The patient denied previous respiratory or cardiac-related diseases. She was administered symptomatic treatments, such as oxygenation, diuresis and so on. However, her symptoms worsen progressively. Then, Abdominal enhancement CT showed uneven thickening of the gastric wall with abnormal enhancement, malignant neoplastic lesions were not excluded. Multiple liver metastases and metastatic lymph nodes on the stomach, abdominal aorta, mesentery, and hepatic portal area locally invaded the adjacent gastric wall (Fig. 3A and B).

**Figure 4.**
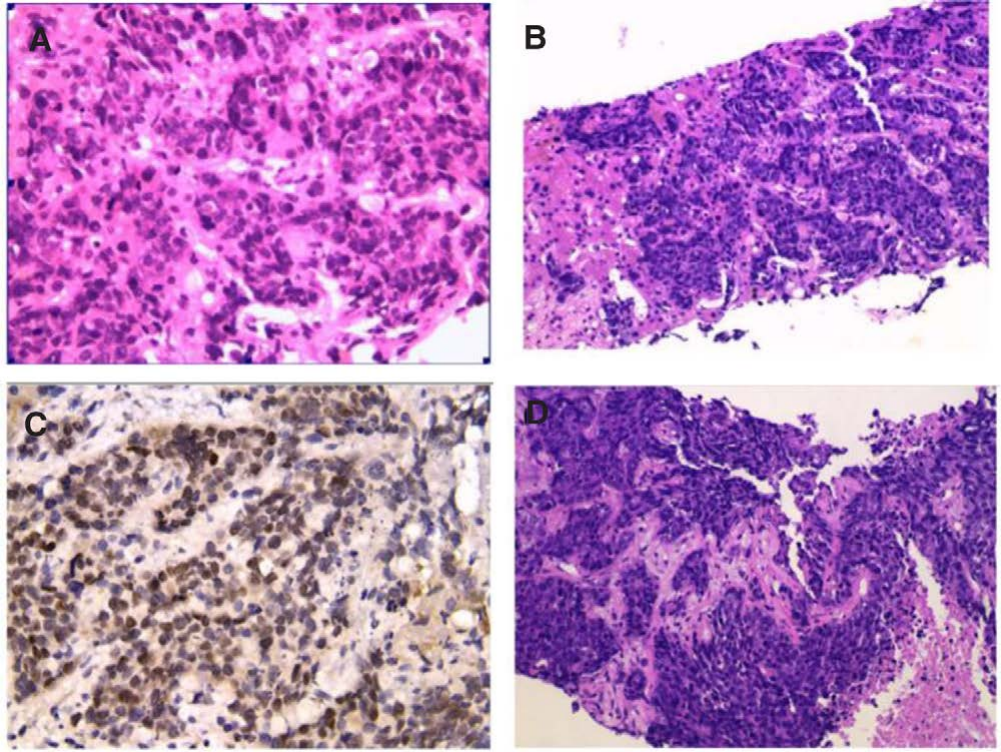
Histopathology. (A) The liver tumor tissue is arranged in nested clusters and beams, with easy-to-see nuclear divisions. (B) Nest-like distribution of cancer cell sheets in normal liver tissue. HE (200×). (C) Carcinoma cells with large deeply stained nuclei, coarse chromatin, easy-to-see nuclear schizophrenic images, tumor necrosis visible in the lower right corner, HE (400×). (D) Immunohistochemical SATB2 staining of the nuclei was positive, suggesting that the cancer cells originated from the gastrointestinal tract (400×).

Liver puncture was done due to patient severe respiratory failure and high risk. Pathological biopsy showed the liver tumor tissue is arranged in nested clusters and beams (Fig. 4A) and the nuclei of cancer cells are large and deeply stained with nuclear schizophrenia under HE staining (Fig. 4B and C). Immunohistochemical results suggest that the cancer cells originate from the gastrointestinal tract (Fig. 4D). After 2 days, the patient condition, including chest tightness and shortness of breath worsened progressively with a progressive increase in NT-pro BNP level to 22,566 pg/mL on retest. There was a continuous decrease in heart rate and oxygen saturation. The patient and her family members discontinued all rescue treatments, including mechanical ventilation, and the patient eventually died. Combining the medical history, symptoms, and related auxiliary examinations, the diagnosis of PTTM was considered clinically.

### 2.2. Case 2

**Figure 5.**
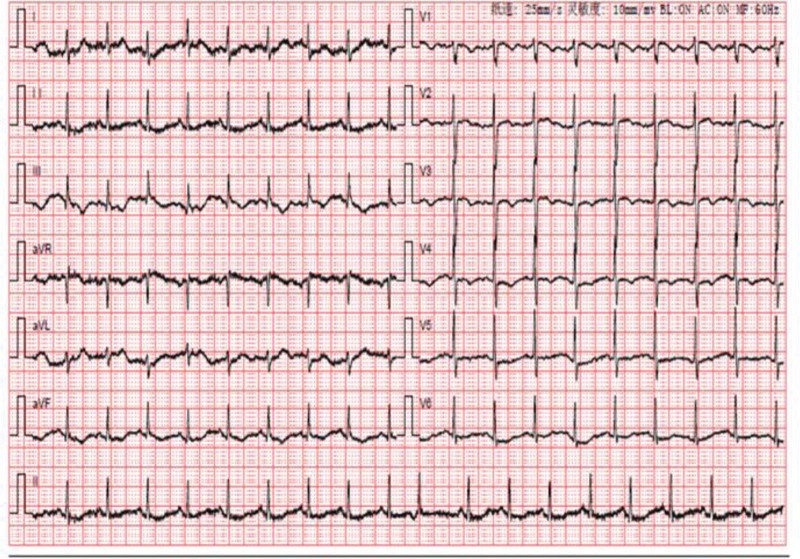
ECG on hospital admission. (1) Sinus tachycardia. (2) ST-T depression. (3) Premature atrial beats. ECG = electrocardiography.

**Figure 6.**
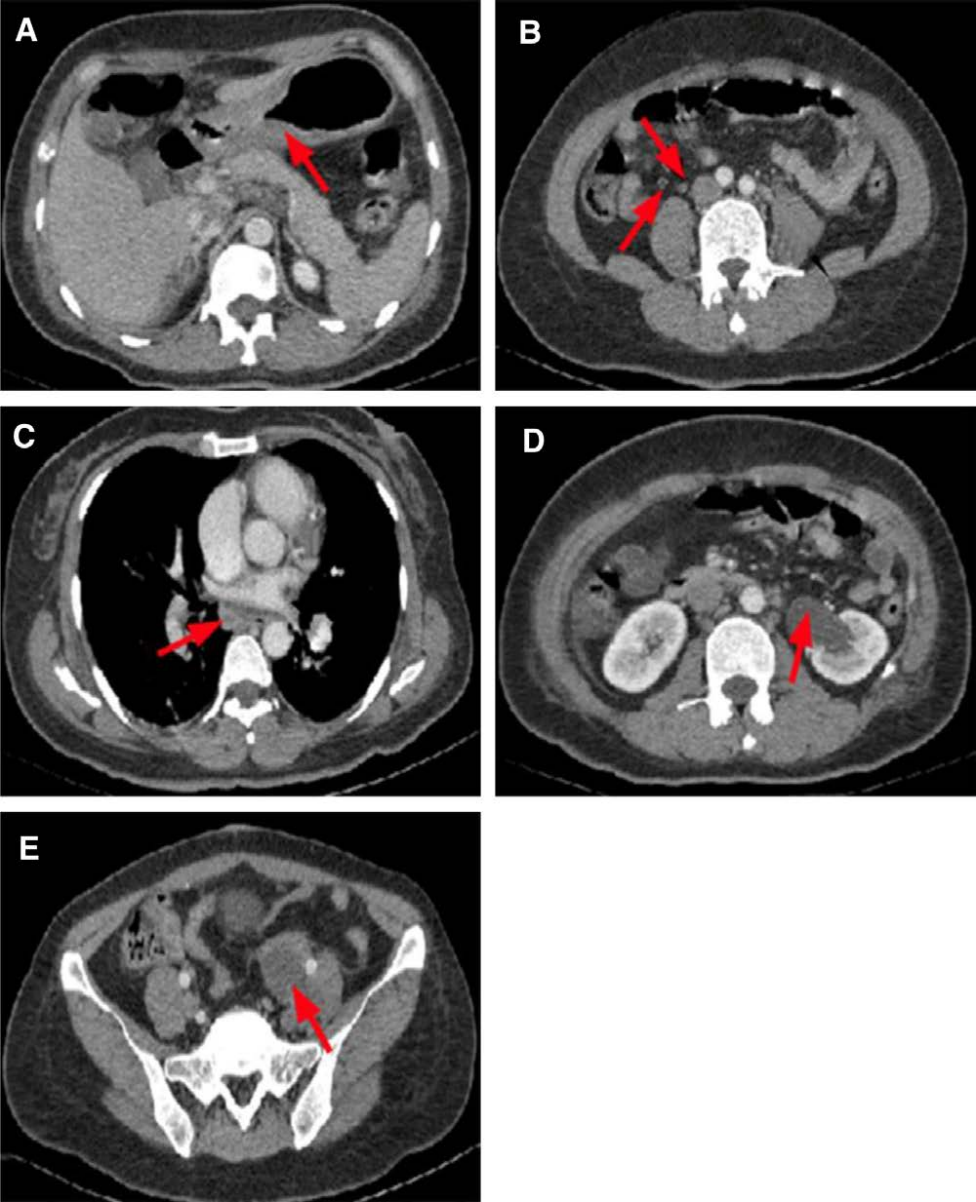
Abdominal enhanced CT. (A) Thickening of the stomach lining. (B) Retroperitoneal lymph node metastasis. (C) Mediastinal lymph node metastasis. (D) Renal pelvis dilatation. (E) Occupying lesion in the left adnexal region.

A 47-year-old woman was admitted to our hospital on November 17, 2021, with intermittent cough and sputum for 2 months, aggravated by chest tightness and shortness of breath for 10 days. Laboratory findings were as follows: WBC, 13.35 × 10^9^/L; NEUT (%), 78.9%; Hb, 139.0 g/L; platelet count, 81 × 10^9^/L; D-D, 7.87 µg/L; and NT-pro BNP, 3790.0 pg/mL. Arterial blood gas analysis showed: PH (T), 7.508; PCO_2_ (T), 22.60 mm Hg; and PO_2_ (T), 67.50% (continuous intranasal high-flow oxygen, oxygen concentration 50%). ECG revealed sinus tachycardia, ST-T depression, and premature atrial beats (Fig. 5). UCG revealed tricuspid regurgitation, pulmonary hypertension (moderate, PASP 54 mm Hg), reduced left ventricular diastolic function, and normal left ventricular systolic function (Table 2). During this period, the patient vomited about 400 mL of black-brown stomach contents and passed 300 g of black paste-like stool, with a positive occult blood test. Disseminated intravascular coagulation (DIC) set showed: D-D, 7.07 µg/L and fibrin degradation products (FDP), 30.58 µg/L. Tumor markers showed CA 199, 1000 ng/mL and CEA, 15.70 ng/mL. Enhanced CT of the abdomen showed local thickening of the gastric sinus wall, Tumors cannot be ruled out (Fig. 6A). Multiple enlarged retroperitoneal and mediastinal lymph nodes were considered as metastases (Fig. 6B and C). Renal pelvis dilatation and Occupying lesion in the left adnexal region (Fig. 6D and E).

**Table 2:** Echocardiogram parameters.

Parameters	Result	Reference value
EF	66.84%	>50%
AAO	33	28–38 mm
AO	23	20–35 mm
LA	33	19–35 mm
RA	44*45	33–41 mm
PA	23	12–26 mm
RVD	23	7–23 mm
TV	3.5	0.3–0.7 m/s

AAO = ascending aorta, AO = aorta, EF = ejection fraction, LA = left ventricle, PA = Pulmonary artery, RA = right atrium, RVD = right ventricle diameter, TV = tricuspidvalve velocity.

Symptomatic treatment such as oxygenation, low molecular heparin anticoagulation and diuretic was administered, and the patient condition continued to deteriorate. The afternoon of the next day, vital signs are unstable. NT-pro BNP, 12,400 pg/mL. The patient died after resuscitation treatment. Combining the clinical manifestations and auxiliary examination, the diagnosis of PTTM was considered clinically.

The 2 patients described in this study were middle-aged women, nonsmokers, and had no previous history of malignancy or other diseases. Both patients, within a short period of time, were presented with progressive dyspnea of unknown origin and acute severe pulmonary hypertension. D-dimer and NT-pro BNP levels were high and blood gas analysis suggested type I respiratory failure. Tumor markers were elevated. No significant intrapulmonary filling defect on CTPA. Malignancy of the stomach could not be excluded on abdominal CT and multiple organ metastases were observed. The first patient liver puncture biopsy pathologically confirmed a malignant tumor of gastrointestinal origin. In summary, acute severe pulmonary hypertension of unknown origin in the 2 patients may be caused by PTTM with progressive heart failure and respiratory failure culminating in death. This case report along with review and analysis of previous literature on PTTM was conducted to raise awareness of the condition among physicians.

## 3. Literature review result

The English search term “pulmonary tumor thrombotic microangiopathy” was used in PubMed to obtain the full-text English literature, and the Chinese search term “pulmonary tumor thrombotic microangiopathy” was used in Wan Fang Data Knowledge Service Platform, China Knowledge Network and Wipu.com to search the Chinese literature. A search of relevant literature published from December 2017 to December 2022 yielded a total of 62 publications (45 in English and 17 in Chinese). The search identified 48 eligible clinical articles with 59 cases of PTTM reported nationally and internationally, the remaining 14 articles were excluded due to recurrence or as they were review articles. Among them, 9 Chinese PTTM articles^[13–21]^ were reported 16 cases; 39 English PTTM articles^[1,22–59]^were reported 43 cases.

### 3.1. Demographic characteristics of all cases

Of the 59 eligible patients with PTTM, 33 (55.9%) were women and 26 (44.1%) were men, aged 29 to 81 years, (with a mean age of 52.9 ± 13.8 years). The primary focus of origin was the stomach in 12 cases (20.3%), followed by the breast in 7 cases (11.9%), the lung in 4 cases (6.8%), tumors from other sites in 11 cases (18.6%) and 25 cases (42.4%) of unknown origin.

### 3.2. Main clinical manifestations

In all cases, 33 cases (55.9%) of respiratory distress, 21 cases (35.6%) of cough and hemoptysis, 1 case (1.7%) of syncope, 3 cases (5.1%) of palpitations, and 1 case (1.7%) of heart failure. All cases had varying degrees of oxygen desaturation and respiratory failure during the course of the hospital stay.

### 3.3. Laboratory and imaging tests

Among all cases, D-dimer levels were increased in 37 cases (accounting for 62.7%), not tested in 21 cases (35.6%), and within the normal range in 1 case (1.7%). Tumor markers were elevated in 21 cases (33.9%), not tested in 36 cases (61.0%), and within the normal range in 2 cases (3.4%). NT-pro BNP was elevated in 14 cases (23.7%), not tested in 41 cases (69.5%), and within the normal range in 4 cases (6.8%). Of the 59 patients, 36 patients (61.0%) underwent chest CT, which indicated nonspecific changes. Among them, 12 patients (33.3%) had ground-glass shadows, 9 patients (25.0%) showed centrilobular nodules and tree bud signs, 16 patients (44.4%) had interstitial changes such as septum thickening and bronchial vascular bundle thickening, and 2 patients (5.6%) had consolidation. Mediastinal lymph nodes were enlarged in 4 patients (11.1%). CTPA was performed in 44 patients (74.6%), and pulmonary artery embolism was ruled out in 42 patients (95.5%). Only 2 patients (4.5%) showed a small peripheral pulmonary artery embolism, which improved completely with anticoagulants. Fifty-seven patients (96.6%) underwent UCG, 40 patients (70.2%) showed enlarged right heart and tricuspid reflux change, 17 patients (29.8%) showed severe pulmonary hypertension, and 40 patients (70.2%) showed mild to moderate pulmonary hypertension (missing accurate values).

### 3.4. Diagnosis

Twenty-eight cases (47.5%) were postmortem with a final diagnosis of PTTM. Thirty-one cases (52.5%) had a clinical diagnosis of PTTM. Among them, 16 Chinese (51.6%) and 11 English cases (35.5%) were analyzed according to the clinical manifestations, laboratory indicators, and imaging examination; 3 English cases (9.7%) showed clinical presentation and evidence of tumor cells in the blood obtained by aspiration cytology of the pulmonary arteries; 1 case (3.2%) of genetic testing showed endothelial growth factor receptor mutation and amplification.

### 3.5. Treatment and prognosis

All patients received different forms of oxygen therapy. Twenty-seven patients (45.8%) were treated with anticoagulants, cortisol hormone, or anticoagulants combined with cortisol hormones and lowering pulmonary artery pressure. Eighteen patients (30.5%) were treated with relevant chemotherapy or targeted therapy for primary or metastatic tumors. Seven patients (11.9%) were treated with other symptomatic support. Four patients (6.8%) were on ECMO, late cardiac life support treatment. No treatment option was mentioned in 12 patients (20.3%). Median survival time since diagnosis for all patients was 9.5 days (CI 5–23.7). Among them, in 8 cases (13.6%), after treatment with pulmonary vasodilators such as endothelin receptor antagonists, their symptoms and pulmonary artery pressure improved, and the survival time improved; their median survival period was 29.5 days (CI 6–57.5), with the longest survival period of up to 106 days. One patient (12.5%) died of respiratory and heart failure within 45h of admission due to rapid progression of the disease. The regression of 2 patients (25.0%) after treatment was ominous.18 Patients (30.5%) treated for their primary disease and treated with radiotherapy and targeted therapy for the primary tumor had an median survival period of 17 days (CI 8.5–14), and least survival period of <1 week. The regression of 4 patients (22.2%) after treatment was ominous.

## 4. Discussion

Tumor-related pulmonary hypertension includes pulmonary vascular damage caused by the tumor itself and pulmonary vascular disease caused by tumor treatment, both of which may lead to pulmonary hypertension and even right heart failure. Pulmonary hypertension associated with the tumor itself is mainly caused by the obstruction of small pulmonary vessels by tumor cells, which activates coagulation and responses such as intimal fibroblast proliferation and pulmonary vascular remodeling, resulting in narrowing or occlusion of the lumen of small pulmonary arteries, usually accompanied by lymphatic vessel dissemination or cancerous lymphangitis. In addition, some mediastinal or pulmonary tumors^[60,61]^ are large enough to compress the main pulmonary artery and the right and left pulmonary trunks or directly invade the proximal pulmonary artery, causing pulmonary stenosis or secondary thrombosis, which can lead to pulmonary hypertension and right ventricular enlargement and eventually right heart failure, but this is less common in clinical practice. Drug-related pulmonary hypertension is primarily caused by tyrosine kinase inhibitors (TKI).

PTTM starts rapidly, with rapid progression and high mortality and can occur in both early and advanced stages of the tumor.^[59]^ Some patients have PTTM as the first manifestation and then diagnosed as a tumor. The 2 cases we reported had no previous history of malignancy; however, they had severe pulmonary hypertension of unknown origin and progressive dyspnea as their first symptoms. After admission, the relevant examination was improved to consider the diagnosis of end-stage gastric cancer with multiple metastasis. Due to the rapid deterioration of the patient condition and the high risk of invasive examination, pathological diagnosis is difficult when the patient is alive. Combined with the clinical manifestations of the patient progressive deterioration, the clinical diagnosis of PTTM was considered.

This should be considered in the differential diagnosis of patients with newly diagnosed pulmonary hypertension and progressively worsening respiratory failure, even in those without a pre-onset cancer diagnosis.^[62]^ A diagnosis of PTTM should be made under the following conditions: Progressive worsening of respiratory distress and hypoxemia. Unexplained elevated pulmonary artery pressure (especially acute severe pulmonary hypertension of unknown origin) or right heart failure. Elevated serum vascular endothelial growth factor (VEGF), D-dimer and FDP levels or a hypercoagulable state without direct or obvious evidence of a pulmonary embolism. CTPA shows pulmonary hypertension with dilated, beaded or tree-like pulmonary arteries without clear signs of pulmonary embolism, chest CT shows central lobular nodules, cloudy peribronchial vessel capillaries, thickened and solid lobular septa, and signs of pulmonary hypertension such as widened pulmonary arteries, enlarged right ventricle, and flattened ventricular septa. Pulmonary perfusion shows multiple wedge-shaped defects in the outer bands of both lungs and mismatched pulmonary ventilation and perfusion images with no obvious evidence of pulmonary embolism. Multiple tumor markers elevated, requiring early consideration of PTTM and diagnosis to increase the chances of saving patients and reducing mortality. However the diagnosis of this disease primarily obtained by autopsy, prebiotic diagnosis is more difficult.

Therapeutically, there is no clear treatment for PTTM, but various treatment options for PTTM as well as potential cancer have been explored. It is well documented that pulmonary vasodilators such as endothelin receptor antagonists, glucocorticoids, warfarin and aspirin, antiproliferative therapies such as platelet-derived growth factor receptor inhibitors (PDGFR) (imatinib) and VEGF receptor inhibitors (bevacizumab) have also been used to target PTTM, particularly in patients with decompensated right heart failure or shock due to pulmonary hypertension.^[63–65]^ In patients with PTTM, inflammation may play a key role in its pathogenesis and several reports suggest that the use of antineoplastic chemotherapy combined with hormonal anti-inflammatory and anticoagulant therapy may reduce patients’ symptoms and lead to improvement of CT lesions in the chest. Although there is no clear evidence that common vasoactive mediators associated with pulmonary hypertension are involved in vascular remodeling in PTTM, they may benefit patients by reducing their pulmonary vasoconstriction.^[14–17,19,21,23,29,30,66]^ In addition, patients who received anticoagulation survived longer after diagnosis than those who did not, suggesting that patients with PTTM have predominantly cancerous thrombotic occlusions in the microvasculature and that early anticoagulation may prolong survival; further data from a large sample size is needed to confirm this. In the 2 cases of unexplained progressive exacerbation of pulmonary hypertension in this study, we could have administered drugs that reduce pulmonary hypertension and perhaps prolonged the survival of the patients. In a case of diagnosed gallbladder cancer with carcinomatous peritonitis reported by Tomioka T et al,^[31]^ the patient died 106 days after treatment with pulmonary vasodilators, soluble guanylate cyclase), and direct oral anticoagulant. A case reported by Ma et al,^[67]^ showed that the combination of apatinib and selexipag for PTTM not only improved clinical symptoms and reduced pulmonary artery pressures but also showed the elimination of lobular septal thickening and gross glassy opacities on CT along with improvement in the patient physical activity tolerance. However, due to unauthorized discontinuation of the patient medication out of hospital, he eventually died. Bevacizumab is a VEGF inhibitor and imatinib is a TKI that blocks phosphorylation of the PDGF receptor and inhibits cell growth. Bevacizumab alone or in combination with imatinib is effective in treating pulmonary hypertension with a reduction in serum VEGF levels, thereby prolonging effective patient survival. One study reported that survival was prolonged after 4 cycles of pemetrexed and cisplatin combination chemotherapy and anti-angiogenic therapy in patients with a prenatal pathological diagnosis of lung adenocarcinoma complicated by PTTM.^[37]^ In this study, the 2 patients had no previous history of malignancy and had developed into terminal stage on admission, which delayed the diagnosis and treatment. And no treatment of the original disease was considered. We gave symptomatic treatment such as oxygen, antispasmodic and anticoagulation, but the patient condition is not in remission. Finally, both died of respiratory and heart failure within a short period of time. However, if the primary disease is detected early, early tumor-specific treatment can reduce the burden of tumor cells in PTTM,^[68,69]^ thereby reducing the stimulation of endothelial proliferation and prolonging patient survival. In conclusion, for patients with PTTM, the early treatment of the primary disease, combined with anticoagulation, pulmonary vasodilator and respiratory support, can prolong the survival period and improve prognosis.

## 5. Conclusion

Both patients were diagnosed with PTTM due to unexplained severe pulmonary hypertension. Currently, as the incidence of cancer is increasing, the frequency of cancer-related lesions, such as thrombotic microangiopathy may also increase. Therefore, PTTM should be considered in the differential diagnosis of primary pulmonary hypertension in non-cancer and cancer patients. However, it is an important cause of tumorous pulmonary hypertension, which can lead to rapidly progressive respiratory failure, heart failure, and death. Prompt diagnosis through a targeted clinical approach could lead to earlier targeted treatment and could improve prognosis.

## Acknowledgments

We thank the General Hospital of Ningxia Medical University, We thank all the patients who consented to donate their data for analysis and the medical staff members who are on the front line of caring for patients.

## Author contributions

**Data curation:** Zhigang Tian.

**Investigation:** Jing Wang, Hai Tan.

**Methodology:** Shaojin Wang.

**Resources:** Jianmei Gao, Lingyan Huang, Xing-Cang Tian, Gang Ma.

**Writing – original draft:** Hua Ma.

**Writing – review & editing:** Shu Xiang Zhang.
